# Genome Sequencing of Methicillin-Resistant and Methicillin-Susceptible *Mammaliicoccus sciuri* from Diseased Animals

**DOI:** 10.1128/mra.00714-22

**Published:** 2022-09-20

**Authors:** Teddy Garcia-Aroca, Stephanie S. R. Souza, Odion O. Ikhimiukor, Michael M. Marcovici, Joshua T. Smith, Sharlene Amador, Colin J. McGonagle, Griffin J. Nye, David B. Needle, Robert Gibson, Cheryl P. Andam

**Affiliations:** a Department of Biological Sciences, University at Albany, State University of New York, Albany, New York, USA; b Broad Institute of MIT and Harvard, Cambridge, Massachusetts, USA; c University of New Hampshire, Department of Molecular, Cellular and Biomedical Sciences, Durham, New Hampshire, USA; d New Hampshire Veterinary Diagnostic Laboratory, Durham, New Hampshire, USA; University of Rochester School of Medicine and Dentistry

## Abstract

Mammaliicoccus sciuri (previously Staphylococcus sciuri) is a frequent colonizer of mammals. We report the draft genomes of a methicillin-resistant strain (2254A) isolated from an armadillo and a methicillin-susceptible strain (6942A) from a cow. Genomes were sequenced using long-read Nanopore sequencing.

## ANNOUNCEMENT

Five Staphylococcus species belonging to the Staphylococcus sciuri group (S. sciuri, S. fleurettii, S. lentus, S. stepanovicii, S. vitulinus) were recently reassigned to the novel genus Mammaliicoccus (1). The M. sciuri group has been hypothesized to carry the evolutionary ancestor of the *mecA* gene (2), which encodes an alternative penicillin-binding protein PBP2a and confers resistance to broad-spectrum beta-lactams (3). M. sciuri is a potential reservoir of resistance and virulence genes that can be acquired by other species (4). It has been reported in humans and animals (5, 6), and has been implicated in disease (7, 8).

Methicillin-resistant M. sciuri (MRMS) strain 2254A and methicillin-susceptible M. sciuri (MSMS) strain 6942A were sampled from animals with confirmed clinical infections (Table 1). Pure isolates were cultured in commercially prepared tryptic soy agar with 5% sheep red blood cells (Thermo Scientific Remel) at 37°C for 24–48 h. Initial species identification was carried out using matrix-assisted laser desorption/ionization time-of-flight mass spectrometry in Bruker Biotyper.

**TABLE 1:** Genome characteristics of the two M. sciuri from diseased animals

Features	MRMS 2254A	MSMS 6942A
Animal source Date of sampling Location No. of reads Bases called Contigs Genome size N50 GC content Genome completeness Genome contamination BUSCO (“bacteria_odb10”) BUSCO (“bacillales_odb10”) CDS Ribosomal RNAs Transfer RNAs Noncoding RNAs (ncRNAs) Antimicrobial resistance genes	Eye of a pet armadillo (order *Cingulata)* April 2018 West Nottingham, New Hampshire, USA 768,000 reads 9,345,898,253 bases One 2,774,130 bp 2,774,130 bp 32.68% 89.5% 4.1% 49.2% 47.6% 3,044 19 58 4 *mecA* (methicillin resistance), *salA* (pleuromutilin-lincosamide-streptogramin A resistance)	Right rear mammary gland of a cow (Bos taurus) September 2018 Durham, New Hampshire, USA 3,384,000 reads 17,538,066,197 bases Two 2,772,237 bp, 32,841 bp 2,772,237 bp 32.54%, 29.1% 96.8% 6.3% 85.5% 83.8% 2,949 19 57 4 *salA*
Heavy metal resistance genes	*arsB* (arsenic resistance)	*asrC* (arsenic resistance), *cadD* (cadmium resistance)
*mecA1* (*mecA* precursor)	Present	Present

Total genomic DNA was isolated using Zymo Quick-DNA high molecular weight Magbead Kit. We used 400 μL of the bacterial cells grown in brain heart infusion broth (BD Difco) containing diluted bacteria and 550 μL of Bashing Bead Buffer in the initial step. We quantified DNA concentration using Qubit fluorometer (Invitrogen) and DNA quality using NanoDrop spectrophotometer (Thermo Scientific).

Sequencing libraries were prepared using the Genomic DNA by Ligation (SQK-LSK110) kit from Oxford Nanopore Technologies (ONT), including the DNA repair step prior to adapter ligation. Long-read sequencing was performed using the MinION platform with R9 flow cells. Sequencing quality was monitored using the MinKNOW v4.5.4 GUI interface. Sequences were base called and demultiplexed using Guppy v5.1.12 (9).

**FIG 1:**
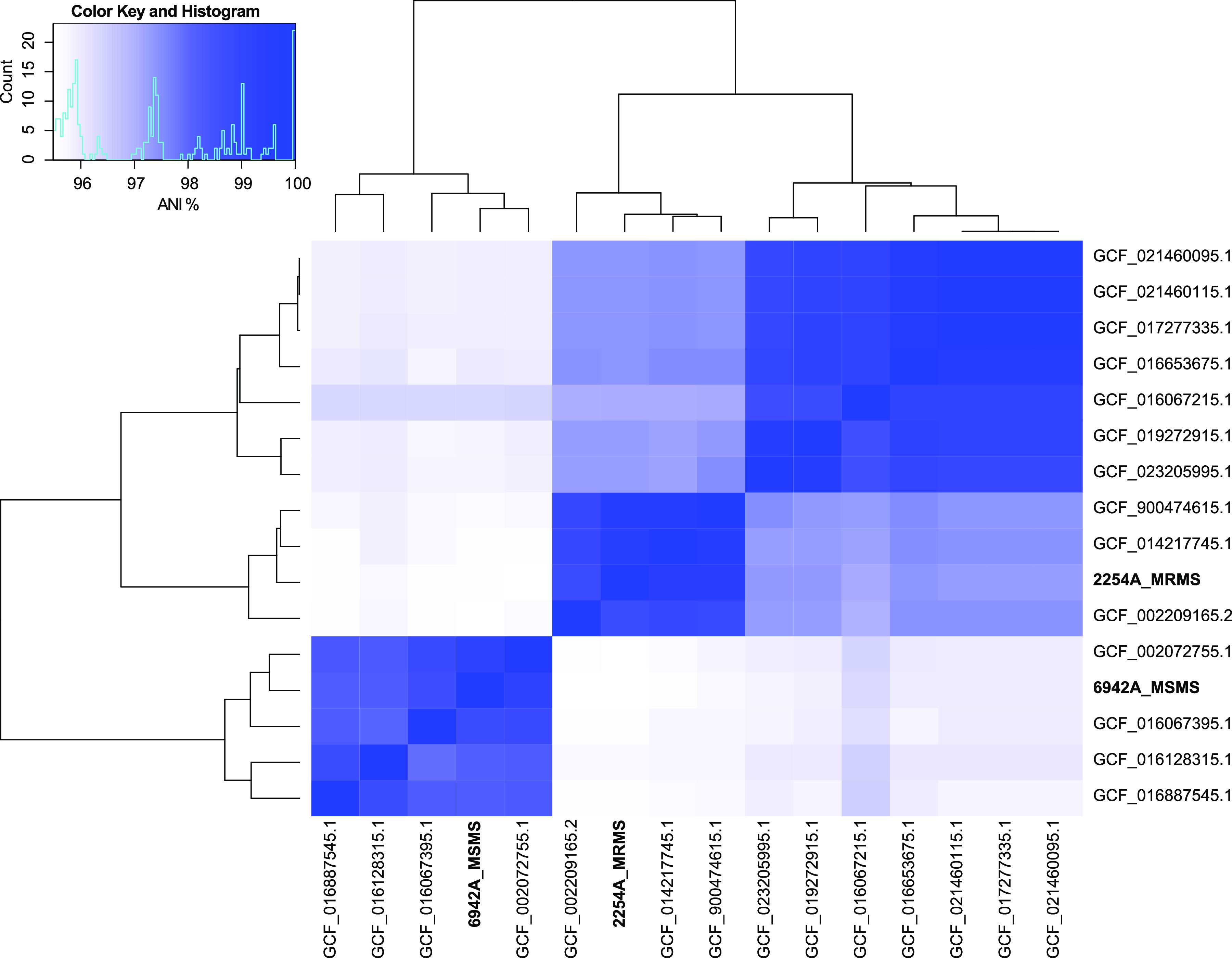
Pairwise comparison of the average nucleotide identity (ANI) between the newly sequenced MRMS 2254A and MSMS 6942A strains, and 14 complete S. sciuri or M. sciuri genomes available in NCBI. Genomes with at least 95% ANI threshold (inset) were considered the same species (19).

Genomes were assembled using open-source scripts from ONT, including the EPI2MELABS wf-bacterial-genomes pipeline, and ran on the nextflow platform (10). Raw fastq files that passed quality controls in MinKNOW were concatenated with fastcat v0.4.10 and assembled with Flye v2.9 (11). Variants, consensus sequences, and polished contigs were obtained with Medaka v1.6.0 (12). Assembly completeness was assessed using BUSCO v5.3.2 (13) and CheckM v1.1.3 (14). Genome contamination, GC content, N50, and number of contigs were estimated using QUAST v5.0.2 (15). Genomes were annotated using the Prokaryotic Genome Annotation Pipeline (PGAP) v6.1 (16) in the National Center for Biotechnology Information (NCBI) (Table 1). Antimicrobial and heavy metal resistance genes were detected using Abricate (https://github.com/tseemann/abricate), AMRfinderPlus (17), and the Comprehensive Antibiotic Resistance Database (18). We used fastANI v1.33 (19) to compare the average nucleotide identity against 14 complete genomes named as either *M. sciuri* or S. sciuri that were available in NCBI as of June 2022 (Fig. 1).

Default parameters were used for all software unless otherwise specified.

### Data availability.

Raw sequence reads have been deposited in the NCBI Sequence Read Archive (SRA) under the BioProject PRJNA851703, with SRA accession numbers SRR19779535 (2254A) and SRR19790864 (6942A). Genome assemblies are available at NCBI under accession numbers CP100353 (2254A) and CP099816/CP099817 (6942A).

## References

[1] Madhaiyan M, Wirth JS, Saravanan VS. 2020. Phylogenomic analyses of the staphylococcaceae family suggest the reclassification of five species within the genus staphylococcus as heterotypic synonyms, the promotion of five subspecies to novel species, the taxonomic reassignment of five staphylococcus species to mammaliicoccus gen. Nov., and the formal assignment of nosocomiicoccus to the family staphylococcaceae. Int J Syst Evol Microbiol 70:5926–5936. doi:10.1099/ijsem.0.004498. 33052802

[2] Rolo J, Worning P, Nielsen JB, Bowden R, Bouchami O, Damborg P, Guardabassi L, Perreten V, Tomasz A, Westh H, de Lencastre H, Miragaia M. 2017. Evolutionary origin of the staphylococcal cassette chromosome mec (SCCmec). Antimicrob Agents Chemother 61:e02302-16. doi:10.1128/AAC.02302-16. 28373201 PMC5444190

[3] Fuda C, Suvorov M, Vakulenko SB, Mobashery S. 2004. The basis for resistance to beta-lactam antibiotics by penicillin-binding protein 2a of methicillin-resistant Staphylococcus aureus. J Biol Chem 279:40802–40806. doi:10.1074/jbc.M403589200. 15226303

[4] Nemeghaire S, Argudín MA, Feßler AT, Hauschild T, Schwarz S, Butaye P. 2014. The ecological importance of the *Staphylococcus sciuri* species group as a reservoir for resistance and virulence genes. Vet Microbiol 171:342–356. doi:10.1016/j.vetmic.2014.02.005. 24629775

[5] Nemeghaire S, Vanderhaeghen W, Argudín MA, Haesebrouck F, Butaye P. 2014. Characterization of methicillin-resistant *Staphylococcus sciuri* isolates from industrially raised pigs, cattle and broiler chickens. J Antimicrob Chemother 69:2928–2934. doi:10.1093/jac/dku268. 25063778

[6] Cirkovic I, Trajkovic J, Hauschild T, Andersen PS, Shittu A, Larsen AR. 2017. Nasal and pharyngeal carriage of methicillin-resistant *Staphylococcus sciuri* among hospitalised patients and healthcare workers in a Serbian university hospital. PLoS One 12:e0185181. doi:10.1371/journal.pone.0185181. 28926634 PMC5605001

[7] Stepanović S, Jezek P, Dakić I, Vuković D, Seifert L. 2005. *Staphylococcus sciuri*: an unusual cause of pelvic inflammatory disease. Int J STD AIDS 16:452–453. doi:10.1258/0956462054093999. 15969784

[8] Chen S, Wang Y, Chen F, Yang H, Gan M, Zheng SJ. 2007. A highly pathogenic strain of *Staphylococcus sciuri* caused fatal exudative epidermitis in piglets. PLoS One 2:e147. doi:10.1371/journal.pone.0000147. 17215958 PMC1764720

[9] Wick RR, Judd LM, Holt KE. 2019. Performance of neural network basecalling tools for Oxford Nanopore sequencing. Genome Biol 20:129. doi:10.1186/s13059-019-1727-y. 31234903 PMC6591954

[10] Di Tommaso P, Chatzou M, Floden EW, Barja PP, Palumbo E, Notredame C. 2017. Nextflow enables reproducible computational workflows. Nat Biotechnol 35:316–319. doi:10.1038/nbt.3820. 28398311

[11] Kolmogorov M, Yuan J, Lin Y, Pevzner PA. 2019. Assembly of long, error-prone reads using repeat graphs. Nat Biotechnol 37:540–546. doi:10.1038/s41587-019-0072-8. 30936562

[12] ONT. medaka: sequence correction provided by ONT Research. https://github.com/nanoporetech/medaka. Accessed 7 June, 2022.

[13] Simão FA, Waterhouse RM, Ioannidis P, Kriventseva EV, Zdobnov EM. 2015. BUSCO: assessing genome assembly and annotation completeness with single-copy orthologs. Bioinformatics 31:3210–3212. doi:10.1093/bioinformatics/btv351. 26059717

[14] Parks DH, Imelfort M, Skennerton CT, Hugenholtz P, Tyson GW. 2015. CheckM: assessing the quality of microbial genomes recovered from isolates, single cells, and metagenomes. Genome Res 25:1043–1055. doi:10.1101/gr.186072.114. 25977477 PMC4484387

[15] Gurevich A, Saveliev V, Vyahhi N, Tesler G. 2013. QUAST: quality assessment tool for genome assemblies. Bioinformatics 29:1072–1075. doi:10.1093/bioinformatics/btt086. 23422339 PMC3624806

[16] Tatusova T, Dicuccio M, Badretdin A, Chetvernin V, Nawrocki EP, Zaslavsky L, Lomsadze A, Pruitt KD, Borodovsky M, Ostell J. 2016. NCBI prokaryotic genome annotation pipeline. Nucleic Acids Res 44:6614–6624. doi:10.1093/nar/gkw569. 27342282 PMC5001611

[17] Feldgarden M, Brover V, Gonzalez-Escalona N, Frye JG, Haendiges J, Haft DH, Hoffmann M, Pettengill JB, Prasad AB, Tillman GE, Tyson GH, Klimke W. 2021. AMRFinderPlus and the Reference Gene Catalog facilitate examination of the genomic links among antimicrobial resistance, stress response, and virulence. Sci Rep 11:12728. doi:10.1038/s41598-021-91456-0. 34135355 PMC8208984

[18] Alcock BP, Raphenya AR, Lau TTY, Tsang KK, Bouchard M, Edalatmand A, Huynh W, Nguyen A-LV, Cheng AA, Liu S, Min SY, Miroshnichenko A, Tran H-K, Werfalli RE, Nasir JA, Oloni M, Speicher DJ, Florescu A, Singh B, Faltyn M, Hernandez-Koutoucheva A, Sharma AN, Bordeleau E, Pawlowski AC, Zubyk HL, Dooley D, Griffiths E, Maguire F, Winsor GL, Beiko RG, Brinkman FSL, Hsiao WWL, Domselaar GV, McArthur AG. 2020. CARD 2020: antibiotic resistome surveillance with the comprehensive antibiotic resistance database. Nucleic Acids Res 48:D517–D525. doi:10.1093/nar/gkz935. 31665441 PMC7145624

[19] Jain C, Rodriguez-R LM, Phillippy AM, Konstantinidis KT, Aluru S. 2018. High throughput ANI analysis of 90K prokaryotic genomes reveals clear species boundaries. Nat Commun 9:5114. doi:10.1038/s41467-018-07641-9. 30504855 PMC6269478

